# The selfish preen: absence of allopreening in Palaeognathae and its socio-cognitive implications

**DOI:** 10.1007/s10071-023-01794-x

**Published:** 2023-05-31

**Authors:** Thomas Rejsenhus Jensen, Claudia Zeiträg, Mathias Osvath

**Affiliations:** Department of Philosophy, Cognitive Science, Cognitive Zoology Group, Lund University, Lund, Sweden

**Keywords:** Allopreening, Autopreening, Dinosaur cognition, Evolution, Palaeognathae, Social cognition

## Abstract

Preening behaviours are widespread in extant birds. While most birds appear to autopreen (self-directed preening), allopreening (preening directed at conspecifics) seems to have emerged only in certain species, but across many families. Allopreening has been hypothesised to reinforce mutual relationships and cooperation between individuals, and to underpin various socio-cognitive abilities. Palaeognathae is a bird group exhibiting neurocognitively plesiomorphic traits compared to other birds. They share many features with non-avian paravian dinosaurs and are thus important for the study of cognitive evolution in birds. Despite this, and the important correlation of allopreening with many complicated social behaviours, allopreening has not been systematically studied in Palaeognathae. Therefore, we examined the preening behaviours in four species of palaeognaths: common ostriches (*Struthio camelus*), greater rheas (*Rhea americana*), emus (*Dromaius novaehollandiae*), and elegant crested tinamous (*Eudromia elegans*). We compared findings with common ravens (*Corvus corax*), a neognath species known for its allopreening and complex social cognition. We found autopreening, but no allopreening, in the palaeognath species, while both autopreening and allopreening was found in common ravens. The absence of allopreening in Palaeognathae suggests an emergence of this behaviour within Neognathae. We contextualise our results in relation to the socio-cognitive underpinnings of allopreening and its implications for the understanding of the evolution of socio-cognitive abilities in non-avian paravian dinosaurs and early birds.

**Supplementary Information:**

The online version contains supplementary material available at 10.1007/s10071-023-01794-x.

## Introduction

Preening the feathers of a conspecific, allopreening, occurs frequently within bird species with long-term pair bonding and parental cooperation (Kenny et al. 2017). Selection for affiliative behaviours, such as allopreening, has been suggested to be associated with the need for cooperation in pair bonded individuals (Kenny et al. 2017; Picard et al. 2020). Increased cooperation and stronger pair bonds between parents might be selected for due to factors such as the altriciality of offspring, predation pressure on nests, and less dense distribution of food (Emery et al. 2007). For many species, allopreening appears to occur within pairs exclusively during the breeding season (Kenny et al. 2017). However, in corvids and parrots, allopreening has gained additional functions, such as a “social currency”, by which individuals can gain benefits in exchange for preening (biological market theory) (Anza et al. 2021). Allopreening is sometimes used between more individuals than just the bonded partner, with some individuals having up to five preening partners (Fraser and Bugnyar 2012; Picard et al. 2020).

It has been hypothesised that when social complexity increases, social challenges that group living animals face, select for larger brains and sophisticated cognitive abilities (e.g., Dunbar 1998; Humphrey 1976; Jolly, 1966). Primates, as well as corvids and parrots, generally live in socially complex groups, at least during parts of their lives. In such groups, social grooming or preening is used to improve and maintain social relationships. For example, ravens are more likely to assist another raven in an aggressive encounter if it had previously been preened by that individual (Fraser and Bugnyar 2012), thus making allopreening an important component of social politics. Indeed, third-party ravens may even interrupt allopreening between other individuals if the resulting bond leads to an increase in social rank for one of the individuals (Massen et al. 2014).

In primates, allogrooming correlates with group size and not body size, emphasizing the importance of the social function of allogrooming, compared to its hygienic functions (Dunbar 1991). Similar to the above-mentioned birds, allogrooming maintains social bonds between individuals in a group (Dunbar 1991). Like ravens, chimpanzees use allogrooming to form alliances and secure support from others during agonistic encounters (Schino 2007), and have also been observed to exchange food items for allogrooming (de Waal 1997).

In both primates and birds, social grooming/preening not only maintains relationships but is also used in post-conflict affiliations, in which previous opponents, or one opponent and a bystander, perform affiliative behaviours after a conflict (Jensen and Osvath 2021). Many primates, corvids, and parrots seek out their previous opponent and mend their relationship via grooming/preening (de Waal and Vanroosmalen 1979; Fraser and Bugnyar 2011; Ikkatai et al. 2016).

Allopreening is widespread among bird taxa and is found in almost all studied families, although with much fewer representatives within Anseriformes (e.g., some ducks and screamers) and Galliformes (e.g., Plain Chachalaca *Ortalis vetula*) (Kenny et al. 2017; Naranjo 1986; Petrie and Rogers 1997; Picard et al. 2020; Stonor 1939), which belong to the neognath groups that are least derived from the palaeognath birds. Hereafter, the prevalence of allopreening increases in species within families that show increased cooperation during offspring care and lower divorce rates (for an overview of allopreening across bird species, see Kenny et al. 2017).

Palaeognathae represents a key group in the avian lineage. Palaeognaths retain many ancestral features that have been lost in the sister taxon, the Neognaths. These ancestral features are found in their early avian ancestors and to an extent in the non-avian paravian dinosaurs. The neuroanatomy of palaeognaths is more like non-avian paravians than any other bird taxa (Kverková et al. 2022; Olkowicz et al. 2016). Ksepka et al. (2020) found that palaeognaths, alongside waterfowl and predatory Telluravians, have the same scaling relationship between body and brain size as non-avian paravian dinosaurs, with other groups sharing other scaling relationships. Additionally, the relative volumes of gross brain areas are similar between non-avian paravians and palaeognaths (Balanoff et al. 2013). Thus, palaeognath birds are optimal models for inferring the cognitive capacities of cretaceous early birds, and to a degree of the non-avian paravian dinosaurs. Furthermore, fossil evidence indicates a reproductive system, and hence a parental care system, in extinct maniraptoran dinosaurs such as oviraptosaurs and troodontids, similar to that of palaeognaths (Moore and Varricchio 2016; Varricchio and Jackson 2016; Varricchio et al. 2008). This reproductive strategy typically involves paternal care of eggs and chicks (although in ostriches, *Struthio camelus*, the dominant female will assist), but with both sexes exhibiting high promiscuity (Valdez 2022). Due to this high promiscuity in both sexes, it is unlikely that any palaeognath species forms stable pairs for life. The young are precocial when hatched and feed on their own without assistance from the parent, lowering the need for more coordinated cooperation between parents during offspring care. This in turn may lower the probability of finding bonding behaviours, such as allopreening.

New studies have begun to provide glimpses into the socio-cognitive capacities of palaeognath birds. Zeiträg et al. (2023a) found social play and play contagion, with an early ontogenetic onset, in greater rheas (*Rhea americana*). Additionally, and more importantly, greater rheas, elegant crested tinamous (*Eudromia elegans*), and emus (*Dromaius navaehollandiae*), exhibit complex gaze following skills, including tracking others’ gaze around barriers, which reveals visual perspective taking (Zeiträg et al. 2023b). This has previously only been observed in primates, dogs and a few songbirds, and is taken to be a key cognitive skill for advanced sociality (Amici et al. 2009; Bräuer et al. 2005; Bugnyar et al. 2004; Butler and Fernandez-Juricic 2014; Met et al. 2014; Range and Virányi 2011; Schloegl et al. 2008; Zeiträg et al. 2022). Along the same lines, the palaeognaths also exhibit “checking back”-behaviours when their expectation of finding a target in a conspecifics’ line of gaze was violated. This is a signature behaviour for the understanding that another individual’s gaze is referring to a target in the environment, which is the foundation or referential communication (Zeiträg et al. 2023b). The “checking back” behaviour has previously only been shown in simian primates, excluding New World monkeys, but may now prove to be widespread in Aves (Zeiträg et al. 2022, 2023b). Despite that palaeognaths are an optimal extant group for making inferences about the early evolution of social cognition in birds and non-avian paravians, and the recent evidence of perspective taking skills and social play in these birds, the presence of fundamental social behaviours, such as allopreening, remains unclear. While the reproductive strategy and social system would suggest low evolutionary pressure for developing allopreening behaviours, their socio-cognitive capacities have recently been shown to be more advanced than previously thought and may indicate more advanced sociality in this group, including a social “currency” in the form of allopreening, than previously believed. Alternatively, the lack of allopreening may indicate that some socio-cognitive abilities can evolve to become quite advanced without the need of complex sociality typically associated with allogrooming birds and mammals.

In this study, we provide the first systematic examination of allopreening in palaeognaths. As palaeognath birds have a precocial young and a reproductive strategy typically not connected to the emergence of allopreening (Kenny et al. 2017), we consider allopreening behaviours to be unlikely in this group. However, due to these birds having recently been shown to possess socio-cognitive skills thought to be quite advanced, we aimed to determine the potential existence of allopreening, and if so to what extent it occurs and during what circumstances. We collected video footage on common ostriches, greater rheas, emus, and elegant crested tinamous. We used common ravens (*Corvus corax*) as an outgroup for comparison, due to their well-known allopreening behaviours (Picard et al. 2020). We discuss our findings in relation to what is currently known about the species’ socio-cognitive capabilities, as well as to the evolution of social cognition in Aves.

## Methods

### Subjects

An overview of species, group compositions, locations, and the distribution of recorded hours between sites and recording periods can be found in Table 1. We observed five species of palaeognath birds. We recorded seven elegant crested tinamous kept by a private owner; eight emus, consisting of one pair kept at North Zealand Bird Park, Denmark, and six individuals kept at Jette’s Ostrich Farm, Denmark; seven common ostriches, one pair kept at Denmarks Bird Zoo, Denmark, and five individuals kept at Jette’s Ostrich Farm, Denmark; and eight greater rheas, three kept at Copenhagen Zoo, Denmark, and five kept at Furuvik Zoo, Sweden. Finally, we also observed six common ravens, one pair and one group of four, kept at Lund University Corvid Cognition Station in Skåne, Sweden. Additionally, the male from the group of three rheas later hatched five chicks. Observational data on both the male and juvenile hatchlings was collected. All animals were kept in outdoor enclosures, were fed daily, and had access to water ad libitum. All individuals, except five, newly hatched juvenile rheas, were sexually reproductive adults. For the pair of emus, the male was tending a nest for roughly half of the observational period. Husbandry at all locations was similar. The animals were not interacted with directly, and all animals kept at zoos, farms, and private owners had been kept together for years and were habituated to visiting guests. The raven group consisted of two first-year siblings which had been added to a group of two females the year prior. We did not observe any stereotypical behaviours in any of our observed animals.

**Table 1:** An overview of species, group compositions, locations, recording periods, and the distribution of recorded hours for this study

Species	Location 1	Location 2	Total (*N*, hours, and days)
Emu	North Zealand Bird Park	Jette's Ostrich Farm	
* N*	2 (pair)	6 (3 males, 3 females)	8
Hours recorded	90.5	54.8	145.3
Number of days recorded	19	11	30
Recording period	19–01-2021 to 26–03-2021	04–10-2021 to 19–10-2021	
Elegant crested tinamou	Private owner		
* N*	7 (6 males, 1 female) (5 males)*		7 (5)*
Hours recorded	26 (20)*		26 (20)*
Number of days recorded	12 (10)*		12 (10)*
Recording period	07–10-2020 to 18–12-2020		
Common ostrich	Denmark’s Bird Zoo	Jette's Ostrich Farm	
* N*	2 (pair)	5 (1 male, 4 females)	7
Hours recorded	68.9	56.3	125.2
Number of days recorded	14	11	25
Recording period	19–07-2021 to 14–09-2021	04–10-2021 to 19–10-2021	
Greater rhea	Furuvik Zoo	Copenhagen Zoo	
* N*	5 (1 male, 4 females)	3 (1 male, 2 females); 6 (1 male and 5 chicks)	8 (13)**
Hours recorded	23.6	28.3; 27.6	79.5
Number of days recorded	8	6; 6	20
Recording period	06–11-2020 to 13–11-2020	31–03-2021 to 05–05-2021	
Common raven	Lund University Corvid Cognition Station		
*N*	2 (pair); 4 (1 male, 3 females))		6
Hours recorded	25.4 (pair); 27.4 (group)		52.8
Number of days recorded	7 (pair); 6 (group)		13
Recording period	17–01-2022 to 28–02-2022		

*The numbers in parentheses signify the data (number of individuals observed, hours recorded, and recording days) of the total that were collected after two individuals died

**The number in parenthesis signifies the total number of individuals observed when including the 5 chicks

These species provided a broad sample of species from the palaeognath clade, representing species from Struthioniformes, Rheiformes, Tinamiformes, and Casuariiformes, largely covering the entire taxon, with the exception of Apterygiformes (kiwi) (Yonezawa et al. 2017). Additionally, data collected on emus and ostriches had differing group compositions as these species may spend time in both groups and in pairs (Bertram 1980; Boland 2003). Common ravens provided an outgroup; they are highly derived, with among the highest number of neurons among birds, and with well-developed social cognition (Boucherie et al. 2019; Olkowicz et al. 2016).

### Data collection

A total of 429 h were video recorded between October 2020 and March 2022 (Table 1). Recordings were collected with up to four cameras filming the enclosure from several angles. A recording session lasted 3–5 h and typically took place continuously between 9:00 and 16:00, alternating between early and late recordings. During the data collection period, two of the seven tinamous died due to circumstances unrelated to this study, leaving the majority of the recordings (20 out of 26 h) with five individuals. Data collection was performed throughout the year and overlapped with breeding seasons for the observed ostrich and emu pairs as well as the group of three rheas. This was done to maximise the potential for detection of allopreening across various social and behavioural contexts.

### Behavioural coding

A detailed list of behaviours and coding definitions is found in the supplementary material. Autopreening was defined as an individual drawing its feathers through their beak. Allopreening included the same behaviours as autopreening but directed at a conspecific. We coded auto- and allopreening for each individual together with the durations of these behaviours when possible (see below). Additionally, we coded the duration of visibility of all individuals that could be consistently identified, as they would sometimes leave the field of view of the cameras. We were able to individually identify all rheas, the pairs of ostriches and emus, as well as the rhea male keeping chicks. For these, we could code each individual’s preening time. It was not possible to reliably identify specific individuals for all tinamous, all ravens, as well as the groups of emus and ostriches. For these groups, a group-level mean of time spent preening was estimated by also coding the number of individuals present on the video at any given time. The mean was then estimated from the number of individuals visible on screen at the time of preening (e.g., if three individuals were preening when five individuals were visible, we divided the sum of the preening duration from the three individuals by five). This provides a reasonable estimate of the average preening rate of a group. The video footage of the male rhea with chicks was coded for allopreening only, as the male’s autopreening behaviour was already coded when he was a member of a group. As the focus of the study was on adult individuals, we only coded for allopreening behaviours of the male towards the juveniles. We did not code any preening behaviours of the juveniles. All behavioural coding was done using Solomon Coder (Peter 2019).

The videos of ostrich and emu groups, as well as all tinamous, ravens, rhea videos, and half of the videos of the emu pair, were all coded by TRJ. The videos of the ostrich pair and half of the videos of the emu pair were coded by CZ. For the inter-rater reliability, TRJ coded 10% of CZ’s coded videos. Inter-rater reliability was calculated using the “irr” package in R (Gamer et al. 2019; R Core Team 2020). Agreement was excellent (two-way mixed-effects intra-class correlation coefficient, based on agreement between single raters: ICC = 0.965, 95% confidence intervals = 0.956–0.972, *P* < 0.001) (Koo and Li 2016).

### Data analysis

We calculated the daily preening budgets (percent time spent preening) of each species. For groups in which individuals were identifiable from each other (ostrich and emu pairs and all rheas), the preening budget for each individual was calculated from the duration of time it was observable, after which a group-level mean was calculated per day observed. For the groups in which individuals could not be individually identified (ostrich and emu groups and all tinamous and ravens), the preening budget for each day observed was calculated from the duration of the videos, as observability had already been taken into account when group-level preening was estimated. Finally, the mean preening budgets for each day of observation were calculated per species.

We also calculated a measure of group observability, defined as the total duration during which allopreening could not occur without being detected (i.e., all, or all but one individual are visible on camera), to give a more accurate measure of the duration in which allopreening had not been observed with certainty. All data analyses were done using R (R Core Team 2020) (Version 4.2.1).

## Results

**Fig. 1:**
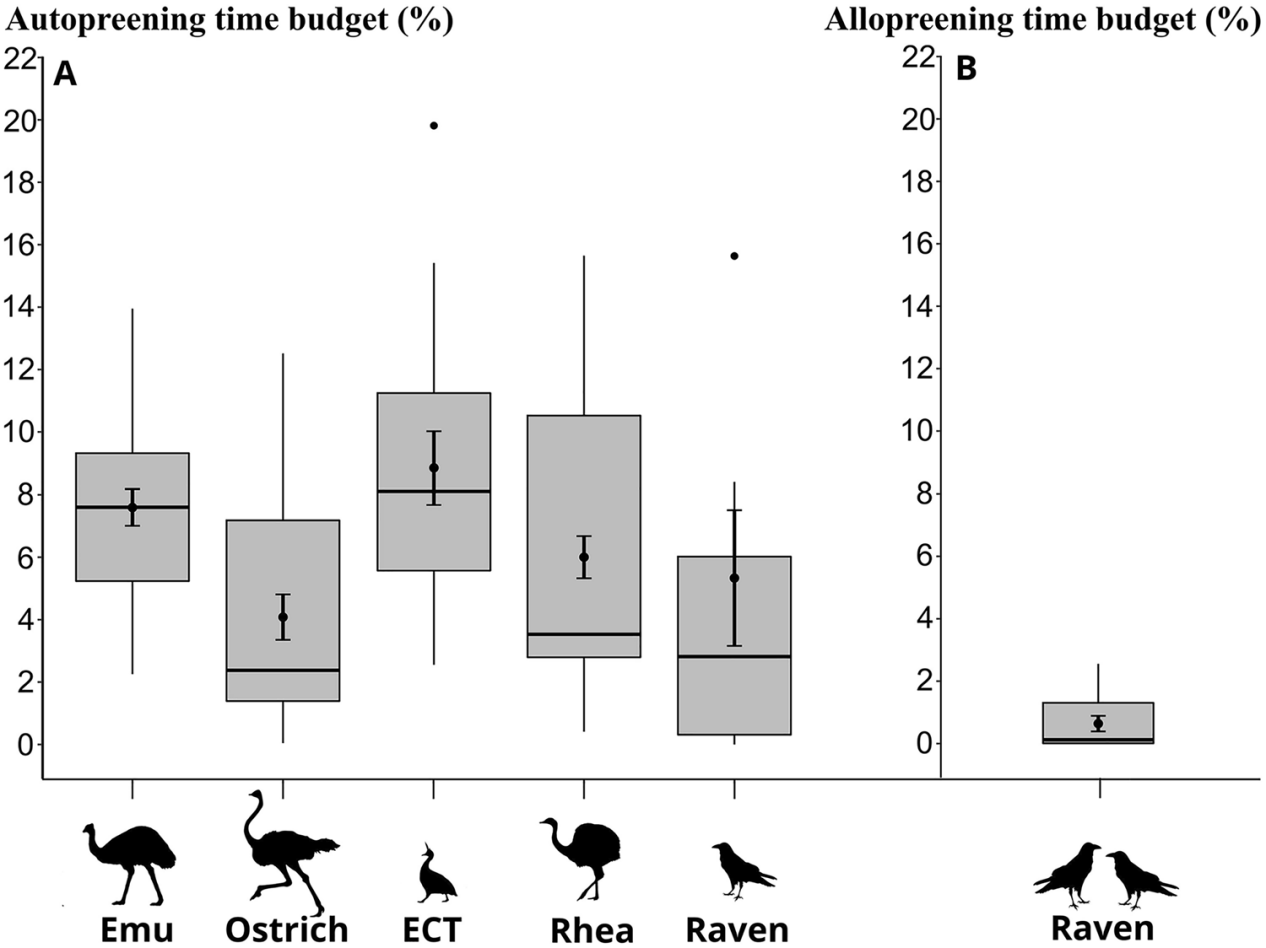
Boxplot of time budgets in percent with associated means (± SE) of **A** autopreening in four species of palaeognath birds (ECT = elegant crested tinamou) and common ravens, and **B** allopreening of common ravens. (Ostrich and raven silhouettes from Phylopic.org, rest by Helena Osvath)

Figure 1 contains a boxplot with added mean daily autopreening and allopreening (when applicable) budgets for all species in this study. In the 429 total hours of video material across the entire observational period, no allopreening was observed in any of the palaeognath species, including the male greater rhea towards its chicks. Only ravens allopreened with a mean daily time budget (mean ± SE) of 0.64% ± 0.25.

Autopreening was frequently observed in all species. The daily autopreening budgets for each species were as follows (mean ± SE): Emu: 7.59% ± 0.59, ostrich: 4.1% ± 0.73, elegant crested tinamou: 8.9% ± 1.17, greater rhea: 6% ± 0.67, common raven: 5.33% ± 2.17.

Group observability, in which all, or all but one, individual in a group could be observed for allopreening, was high: greater rheas: 47.3 h, 90% of total video duration (+ 23.8 h visibility of the male with chicks, 86% of total video duration); elegant crested tinamous: 23.9 h, 88% of total video duration; ostriches: 91.5 h, 73% of total video duration; emus: 128.1 h, 88% of total video duration; common ravens: 46.1 h, 87% of total video duration.

## Discussion

Allopreening was not observed in any of the palaeognath species in this study. The ravens showed allopreening, which was expected. The allopreening budgets found in this study are in agreement with data from other allopreening species. Picard et al. (2020) examined allopreening in 12 species of parrots and corvids and found a median percentage of total time spent allopreening of 1%, with the majority of observations being below this value. Allopreening time budgets in common ravens of their study was at 0.66% (Picard et al. 2020). This is higher than the budget of 0.12% observed in the present study. We believe that this difference can be explained by the group composition of the four ravens in our study, consisting of two females and two first-year siblings with quite low allopreening rates, as they were still learning how to socialise within the group. The three groups examined by Picard et al. (2020) consisted of eight-to-ten individuals. Importantly, allopreening was still observed in both groups of ravens in our study, despite the low rates, while allopreening was never observed in any of the palaeognath species. This is in spite of the breeding behaviours in the ostrich pair, emu pair as well as the rhea group containing three individuals. Specifically, the ostrich pair was observed mating twice, the emu pair was observed mating once and the male was tending a nest for half of the observational period. The rhea male from the group of three was observed courting the females on several occasions, and mating as well as egg-laying by the females took place during the observational period.

All species in this study autopreened regularly. The rates of autopreening found in the palaeognaths were comparable to preening reported by other observational studies on these species. Our mean autopreening budget of 6% in greater rheas is in line with the findings of de Azevedo et al. (2010), who reported an autopreening budget of 4.1% across their study period. Buclaw and Szczerbińska (2017) found preening rates in emus varying from 11 to 19% of their daily time budget depending on seasons, which is quite high compared to our average of 7.6%. However, it is worth noting that they also included head scratching and bathing in puddles in this category, which could have significantly inflated the actual preening rates. We also note that the male in our observed pair of emus spent half of the observation time incubating a nest and thus may have autopreened less during this time, but this did not have a noteworthy effect on the total preening budget of the species. We are not aware of any other studies on the preening rates of elegant crested tinamous. However, Garitano-Zavala et al. (2003) have studied the behaviours of ornate tinamous (*Nothoprocta ornata*) and found no allopreening, but seasonal variation in autopreening rates between 1% (September) and 8.5% (March). Cromberg et al. (2007) recorded preening rates in various group compositions of the closely related red-winged tinamou (*Rhynchotus rufescens*) (Bertelli et al. 2002), during the breeding period, which averaged at 13.5%. This is higher than the 8.9% in our elegant crested tinamous, but within the observed variation. It is also of note that 20 of our 26 h of footage of the tinamous only contained males, while the last 6 h contained six males and one female. This may have affected the preening rates of our tinamous as there was no breeding period. Given the group composition and observation time on the elegant crested tinamous observed in this study, we are cautious to conclude anything definitive on their preening behaviours. For common ostriches, McKeegan and Deeming (1997) reported mean autopreening time budgets of 1.5–5%, which is in agreement with our observations. Surprisingly, the authors also mentioned allopreening at 0.1–0.9% mean rates. However, it is likely that regular pecking of other individuals, a common behavioural problem in captive ostriches (Reischl and Sambraus 2003), has been misidentified as allopreening. These two behaviours are, however, very different, with the pecking of conspecifics being more akin to behaviours when pecking the ground, while allopreening (and autopreening) involves a movement in which the feathers are drawn through the beak. Given the definition of preening given by the authors, pecking at conspecifics could be included in their definition of allopreening. This pecking of conspecifics was also observed in our recordings but was not categorised as allopreening. If, indeed, McKeegan and Deeming (1997) found true allopreening, it is incredibly rare, as it was not found in our 125 h of ostriches observed, with all or all but one individual observable for 73% of the total video material. For these reasons, it is unlikely that these instances represent cases of true allopreening. Alternatively, this pecking might represent a rudimentary form of social grooming behaviour.

North Island black kiwi (*Apteryx mantelli*) autopreen and appear to exhibit a form of social grooming during mating, in which the male will probe the females feathers with his beak repeatedly. This resembles allopreening, but has only been described anecdotally in relation to the mating act itself, and is otherwise not found (Cunningham and Castro 2011), even though some kiwi show social monogamy due to ecological constraints (Taborsky and Taborsky 1999). We therefore urge others to more systematically investigate kiwi birds, which is the only major group of palaeognaths lacking from our material.

Likewise, southern cassowary (*Casuarius casuarius*) males may lightly peck a female’s body and head during courtship before mating, even though they are completely solitary outside of the reproductive season (Schmitt and Ly Vere 1981). It is possible that this tactile stimulation during courtship and mating may provide the first evolutionary step towards more frequent and well-developed allopreening, first expanded to cover the breeding season, providing affiliative benefits during offspring care, and then later evolved to also function as a social currency in socially complex species.

Interestingly, even within the non-volant palaeognaths of this study (emus, common ostriches, and greater rheas), autopreening rates were similar to those of common ravens, despite a lack of need for pristine feathers for flight. This suggests that the functions of autopreening cannot be fully explained feather maintenance for enabling flight. Other functions, such as the removal of dirt, other particles and ectoparasites, as well as applying preen gland oil, might be the primary function of this behaviour (Clayton et al. 2010). Moreover, this indicates that the evolutionary roots of autopreening are not tied to the emergence of flight—if it is not a vestigial behaviour in the secondarily flightless animals—but that this behaviour could have emerged with the evolution of the earliest integumentary feathers. When exactly the earliest integumentary feathers emerged is still debated. Fossil evidence shows early monofilamentous feathers relatively frequent in Coelurosauria and later clades (Benton et al. 2019; Rauhut et al. 2012; Xu and Guo 2009). It has even been suggested that the fine pelt on pterosaurs consists of monofilamentous feathers (Yang et al. 2019), pushing the emergence of feathers back to 250 million years ago. This has, however, been disputed by others (Unwin and Martill 2020) and will require more studies to provide further evidence.

Alternatively, autopreening could have become necessary only when complex feather structures evolved. If so, one can reasonably assume that many maniraptoran dinosaurs, and likely all non-avian paravian dinosaurs, preened to maintain their complex feather structures. At this point in evolution, feathers had rachis and vanes and resembled feathers known from extant birds. Some species even had feather types that are not represented in any living birds today (Xu and Guo 2009; Xu et al. 2010). However, given how autogrooming is widespread in many mammals with fur that have less complex structures than feathers, it is likely that even monofilamentous feathers would require some level of maintenance. It is thus quite likely that dinosaurs with feathers preened themselves.

The lack of allopreening in palaeognaths has some implications for the perspective on the evolution of some socio-cognitive skills in Aves. Allopreening is typically associated with stable pair bonds across long timescales and cooperation in most birds (Kenny et al. 2017), but is also observed in relation to more complex behaviours such as reconciliation and social politics in corvids and parrots (Fraser and Bugnyar 2011, 2012; Ikkatai et al. 2016; Massen et al. 2014). One could expect that allopreening and its use would co-vary with socio-cognitive complexity. According to the social brain hypothesis, complex social structures would select for increasingly more sophisticated social cognition to better navigate the social environment, such as visual perspective taking (Emery 2000), affiliative behaviours for mending damaged relationships (Jensen and Osvath 2021), or social play for acquiring social norms and competences (Palagi 2018).

However, evidence is accumulating that palaeognath birds possess several sophisticated socio-cognitive skills, e.g., visual perspective taking, while apparently not engaging in any form of allopreening behaviour. Visual perspective taking and social play, including play contagion, has evolved in palaeognaths despite the lack of complex social structures or deep social bonds (Zeiträg et al. 2023b). This indicates that socio-cognitive skills are not clearly linked to such structures or bonds, and the allopreening behaviours associated with them. Thus, such skills may have evolved much earlier in birds than strong social bonding, allopreening, and associated behaviours. While sociality is likely an important factor in the evolution of complex cognition, as stated by the social brain hypothesis, evidence is accumulating that its role as the main driver of brain evolution is not supported (Acedo-Carmona and Gomila 2016; Chambers et al. 2021; Finarelli and Flynn 2009; Hooper et al. 2022; Kverková et al. 2018; Powell et al. 2017), and perhaps not even for well-developed social skills. Decreasing the uncertainty about the world through gathering information from social sources is highly adaptive and likely beneficial to animals independent of their social system (Schmidt et al. 2010). This notion is supported by a variety of studies on social cognition in purportedly asocial reptiles. For example, gaze following and social learning have been found in the red footed tortoise (*Geochelone carbonaria*) (Wilkinson et al. 2010a, b). These studies further challenge the notion, that social systems more complex than merely tolerating other individuals, are required for the evolution of some key aspects of social cognition. The ability to discriminate between individual conspecifics based on individual identity cues, the so-called true individual recognition, has been shown in the territorial but otherwise asocial Iberian wall lizard (*Podarcis hispanica*) based on scent (Carazo et al. 2008; Tibbetts and Dale 2007). True individual recognition is considered the highest level of individual recognition, more sophisticated than class-level recognition (recognising an individual to belonging to a class of individuals), as well as distinguishing between categories of conspecifics (e.g., offspring vs. others) (Tibbetts and Dale 2007; Wiley 2013). True individual recognition lays the foundation for the evolution of many other socio-cognitive abilities that require the attribution of experiences to conspecifics. For these reasons, one could expect more socio-cognitive abilities to be adaptive for non-social species.

Research on social behaviours in palaeognaths is important for understanding the evolution of social cognition and behaviours in non-avian dinosaurs and early birds. As the neurocognitively most plesiomorphic extant group of birds that split from other birds before the K-Pg extinction event (Yonezawa et al. 2017), they share many similarities with the non-avian dinosaurs, e.g., similar scaling relationship between brain and body and similar reproductive systems (Ksepka et al. 2020; Moore and Varricchio 2016; Varricchio et al. 2008). This suggests that allopreening had not evolved in non-avian paravians, but emerged later in the avian lineage, likely alongside biparental care (Kenny et al. 2017). Indeed, allopreening has been described in species within Galloanserae, the first neognath lineage to split from the rest of neognath birds (Kenny et al. 2017; Petrie and Rogers 1997; Stokes 1967). Social interactions involving grooming as a social currency, the way we see in primates, corvids, and parrots, would thus likely not have been present in non-avian paravians, unless independently evolved in some taxa. However, this does not necessarily mean that no other forms of social currency could have evolved at some point within the non-avian dinosaur lineage, though currently no evidence of this exists.

Taken together, the absence of social grooming in palaeognaths suggests that their social structures do not utilise allopreening as a social currency to maintain relationships between individuals. It is likely that the need for social currencies evolved alongside the increased need for strong social relationships, either primarily between partners as in birds, or between many individuals in a group setting as in primates (Picard et al. 2020). However, our findings hence suggest that many advanced socio-cognitive skills may have evolved in non-avian paravians without strong social bonds. Further studies on social cognition in palaeognath birds will uncover what social skills that might have been present in non-avian paravians and early birds, and form a baseline for further studies into the evolution of avian socio-cognitive skills.

## Supplementary Information

Below is the link to the electronic supplementary material.

Supplementary file1 (DOCX 14 KB)

## Data Availability

Data and code used in this article are available on Figshare: https://doi.org/10.6084/m9.figshare.21731369.
