# Supplementary material for: The selfish preen: absence of allopreening in Palaeognathae and its socio-cognitive implications
Source: Anim Cogn. 2023 May 31;26(5):1467–76. doi: 10.1007/s10071-023-01794-x (PMC10442270; doi:10.1007/s10071-023-01794-x)
Supplement: Supplementary file 1 — Supplementary file1 (DOCX 14 KB) [file 10071_2023_1794_MOESM1_ESM.docx]

**The selfish preen: Absence of allopreening in Palaeognathae and its socio-cognitive implications**

**Animal Cognition**

Thomas Rejsenhus Jensen^a^*, Claudia Zeiträg^a^ and Mathias Osvath^a^

^a^*Department of Philosophy, Cognitive Science, Cognitive Zoology Group, Lund University, Sweden*

*Corresponding author: [thomas_rejsenhus.jensen@lucs.lu.se](mailto:thomas_rejsenhus.jensen@lucs.lu.se)

**Supplementary materials**

**Coding definitions**

Duration and frequency of auto- and allopreening, as well as measurements of observability of individuals, was extracted from video footage using Solomon Coder (Version 19.08.02). The indicators and behaviours were defined as follows:

Observable (Focal) [Duration]: The time in which the head of the focal individual and its actions are clearly visible in the video.

Observable (Group) [Duration]: The time in which the head and its actions of all or all but one individual is clearly visible in the video.

Autopreening [Duration]: Time spent autopreening defined as the duration from the beak touching the body to preen until the head returns to its normal vigilant position. If 3 seconds had passed with no autopreening occurring, the behaviour was marked as ended (without including the 3 second period in the preening duration).

Allopreening [Duration]: Time spent allopreening defined as the duration from the beak touching a conspecific’s body to allopreen until the head returns to its normal vigilant position. If 3 seconds had passed with no allopreening occurring, the behaviour was marked as ended (without including the 3 second period in the preening duration).

Individual (1-7) [Duration]: The number of individuals with their head and its actions visible on the screen at any given time.
